# Historic Variations in Winter Indoor Domestic Temperatures and Potential Implications for Body Weight Gain

**DOI:** 10.1177/1420326X11425966

**Published:** 2013-04

**Authors:** A. Mavrogianni, F. Johnson, M. Ucci, A. Marmot, J. Wardle, T. Oreszczyn, A. Summerfield

**Affiliations:** a UCL Energy Institute, University College London, London, UK; b Department of Epidemiology and Public Health, University College London, London, UK; c The Bartlett School of Graduate Studies, University College London, London, UK

**Keywords:** Central heating, Housing, Indoor temperature, Thermal comfort, Obesity, Weight gain

## Abstract

It has been argued that the amount of time spent by humans in thermoneutral environments has increased in recent decades. This paper examines evidence of historic changes in winter domestic temperatures in industrialised countries. Future trajectories for indoor thermal comfort are also explored. Whilst methodological differences across studies make it difficult to compare data and accurately estimate the absolute size of historic changes in indoor domestic temperatures, data analysis does suggest an upward trend, particularly in bedrooms. The variations in indoor winter residential temperatures might have been further exacerbated in some countries by a temporary drop in demand temperatures due to the 1970s energy crisis, as well as by recent changes in the building stock. In the United Kingdom, for example, spot measurement data indicate that an increase of up to 1.3°C per decade in mean dwelling winter indoor temperatures may have occurred from 1978 to 1996. The findings of this review paper are also discussed in the context of their significance for human health and well-being. In particular, historic indoor domestic temperature trends are discussed in conjunction with evidence on the links between low ambient temperatures, body energy expenditure and weight gain.

## Introduction

It has often been argued that the five decades since the 1960s have seen a significant rise in indoor temperatures. This has been partly attributed to a shift of cultural norms towards thermal comfort [1]. In his historical analysis of the construction of thermal comfort standards, Healy [2] discusses the trends underlying the increased occupant preference for “thermal monotony”, which is maintained via scientifically delineated norms of thermal comfort that configure a standardized, homogenous “comfort zone”. The thermal homogenization of indoor environments across the years was mainly driven by the uptake of central heating [3–6] and air conditioning [7–12] that deliver uniform thermal conditions and are commonly linked to a subsequent rise in occupant comfort expectations. Furthermore, it has been suggested that the rise in indoor temperatures is strongly correlated with the increased wealth of modern societies, as well as low fuel prices and greater efficiency of building fabric and building systems in newer buildings.

In recent years, a considerable amount of literature has been published on thermal indoor environments and associated comfort expectations in various countries. Nevertheless, the generalisability of the research is problematic because of the lack of longitudinal studies across nationally representative samples of buildings. A trend of rising winter indoor temperatures in recent years in industrialised countries worldwide is often mentioned [1,6,13,14], potentially partly driven by climate change-induced rises in external temperatures. Unfortunately, however, this claim usually relies on indirect evidence or modelled data. Other authors have also commented on the lack of reliable empirical data on indoor temperatures in large housing samples [13,15,16]. To our knowledge, no summary or comparative analysis of the existing data on indoor winter temperatures in industrialised countries has been produced to date.

Understanding the dynamics of indoor climate change is crucial for the impact assessment of internal environmental conditions on human health and well-being. For instance, the reduced exposure to ambient temperature variability and the increased time spent in the thermoneutral zone (TNZ) have been identified as potential contributors to the increase in obesity during the last century [17,18]. So far, this hypothesis remains untested and the epidemiological evidence is scarce. The TNZ can be defined as the ambient temperature at which the human body does not have to initiate physiological processes in order to maintain thermal homeostasis. For naked humans this is said to be 25–27°C [19], although this is affected by a number of individual and environmental factors such as age, sex, sleeping or waking state, activity level, body composition and wind chill.

The present review forms part of a multidisciplinary study of the impact of changes in the domestic thermal environment on weight gain. Within the context of this study, a parallel review [20] has documented the evidence for metabolic responses to mild cold compared with a thermoneutral environment. The main aim of this paper is to evaluate changes in historic winter indoor residential temperatures in industrialised countries. It also provides a brief overview of evidence on the links between low ambient temperatures, metabolic energy expenditure and weight gain.

This review, therefore, seeks to address the following research questions:

1. Is there evidence of a rise in indoor domestic winter temperatures to which individuals are exposed in the last decades in industrialised countries?
2. How are indoor domestic winter temperatures likely to change in future, taking into account saturation effects, climate change and human adaptability?
3. Is there a biologically plausible link between the reduced exposure to mild cold, body energy expenditure and weight gain? If so, how does this compare with indoor temperature trends?

An account is given of recorded changes in indoor temperatures with a focus on domestic environments by summarising relevant existing household surveys carried out in industrialised countries around the world. This review focuses mainly on the United Kingdom as a case study, where data were more readily available. Data from other countries are presented for comparison, including the United States, a number of Scandinavian and Asian countries, and New Zealand. Most of the available data cover the period from the original oil crisis in the 1970s onwards. Unfortunately, there are few studies with longitudinally monitored summer indoor temperature data because until recently, summertime performance of buildings was not a major concern in the mostly heating-dominated countries examined. As a result, this review focuses on the winter indoor conditions.

Evidence on the biological plausibility of a link between decreased cold exposure and adiposity is presented in brief, with a focus on the impact of mild cold on energy expenditure and thermogenic capacity. (The thermogenic capacity of a mammal incorporates the basal metabolic rate (BMR), as well as nonshivering thermogenesis (NST) and shivering thermogenesis (ST) mechanisms.) An attempt to estimate the potential magnitude of such an effect was made by superimposing estimates of decreases in human energy expenditure in response to ambient temperature rises on the corresponding mean domestic indoor temperature increase for the United Kingdom across two decades. Understanding trends in the levels at which people heat their homes is crucial: it could inform government policies aiming to reduce household energy, as well as the impact assessement of indoor environmental conditions on human health – including, as for the purposes of this study, potential body weight gain.

## Evidence of Changes in Indoor Domestic Winter Temperatures

### United Kingdom

The first extensive nationwide survey of domestic winter indoor temperatures in the United Kingdom was the 1978 UK Nation Field Survey of House Temperatures, conducted by Hunt and Gidman from February to March 1978, in 901 houses [21]. A combined approach of spot-reading measurements and occupant interviews was adopted. They recorded a mean dwelling temperature of 15.8°C (18.3°C in the living room, 16.7°C in the kitchen and 15.2°C in the warmest bedroom of the dwelling). According to the authors, a quarter of the visits to the houses were made in the morning (before 1300 h), a quarter during the afternoon (between 1300 h and 1800 h) and half during the evening (after 1800 h). Importantly, the majority of the visits (85%) were made on a weekday and the rest during the weekend.

Extensive longitudinal evidence of an increase in desired winter thermal comfort levels, as observed two decades later, was presented in the 1996 Energy Report of the English House Condition Survey [22]. Temperature spot measurements were carried out mostly during the day on both weekdays and weekends in nationally representative samples of the English domestic stock of approximately 16,000–17,500 dwellings during the 1986 and 1996 English House Condition Surveys. It appears that between 1986 and 1996, 2 years with relatively similar external climatic conditions, the mean living room temperature increased by 0.9°C (19.1°C in 1996) and the mean hall temperature (a relatively good proxy of mean dwelling temperature [21,22] by 1.6°C (17.9°C in 1996).

The most recent UK national level survey was conducted from July 2007 to February 2008 within the context of the Carbon Reduction in Buildings (CaRB) research project [16]. The study drew on a sample of 427 nationally representative dwellings and included both monitored winter temperatures in living rooms and self-reported central heating thermostat settings. The actual indoor temperature measurements were used to produce estimates of the thermostat settings, which were subsequently compared with respondent-reported settings for the subsample of houses that were served by gas/oil-fired central heating systems and comprised 84% of the CaRB sample (358 houses). For each heating day, the thermostat setting was estimated to be equal to the *maximum* living room temperature on that day. Due to methodological differences, these values should not be directly compared with the previously mentioned UK indoor temperature spot measurement studies. It was observed that participants tended to report much lower thermostat settings than the actual temperatures (18.7°C and 19.1°C reported from the participants compared to 21.3°C and 21.1°C estimated from the logger readings in the living room and in the hall, respectively).

In addition to the above, the Building Research Establishment (BRE)’s Housing Model for Energy Studies (BREHOMES) was used to produce broad estimates of internal dwelling temperatures from 1970 to 2006 [6]. The core calculation engine of BREHOMES is the BRE Domestic Energy Model (BREDEM). In brief, BREDEM algorithms were used to calculate heat losses of different dwelling types relying on available statistical data where possible. Subsequently, the percentage of fuel used for space heating was estimated by breaking down the aggregate total delivered energy figure for the domestic sector into different uses. The mean internal temperature was then calculated using heat balance equations and calibrated to top-down national level statistics of energy consumption (the “reconciliation procedure”). According to the authors, the model is run once and its estimates for the various dwelling types are summed up based on the occurrence of each type in the stock. This aggregate figure is then compared to the aggregate energy consumption figure provided by the Digest of United Kingdom Energy Statistics (DUKES) [23] for the corresponding year. The demand temperatures are then adjusted and the calculations are repeated until perfect agreement is reached between the model and the DUKES data. This suggests that all the uncertainty in modelling results is attributed to a rise in demand temperatures, whereas in reality there are many uncertainties in the model. According to these estimates, the average winter internal temperature has increased by 5.7°C between 1970 and 2006 despite the fact that the 2 years were characterized by similar external climatic conditions (the difference between the mean external temperature in 1970 and 2006 in Great Britain was only 1°C). The authors attributed the increase principally to the larger proportion of centrally heated homes. The modelled indoor temperature values are significantly lower than those reported in the English House Condition Surveys [22] or any of the other UK studies but this discrepancy should be mainly attributed to the caveats of the method explained above.

Existing UK empirical and modelled data are summarised in Table 1 below. The BREHOMES modelled data covers the period 1970–2006; only a small sample of this data is presented in Table 1 below for comparison purposes. The full data set can be found elsewhere [6].

**Table 1:** Historic data on winter indoor air temperatures across two decades based on statistically representative national household surveys in the UK; Sources: [6,16,21,22]

Authors	Year	Number of houses, N	Space	Sample mean, μ	Standard deviation, Σ
Spot measurement method					
Hunt and Gidman [21]	1978	901	Dwelling	15.8°C	2.9
			Living room	18.3°C	3.0
			Hall	15.6°C	3.2
			Kitchen	16.7°C	3.1
			Warmest bedroom	15.2°C	3.3
DETR [22]	1996	16,000–17,500	Living room	19.1°C	2.7
			Hall	17.9°C	3.4
			Circulation space	17.7°C	4.2
			Kitchen	18.1°C	3.0
Main bedroom	18.5°C	2.8
			Other bedroom	17.0°C	2.6
			Bathroom	15.0°C	5.0
Estimated thermost settings method					
Shipworth et al. [16]	2007	358	Living room	21.3°C	2.0
			Hall	21.1°C	2.6
Occupant reported thermost settings method					
Shipworth et al. [16]	2007	358	Living room	18.7°C	3.4
			Hall	19.1°C	3.0
Modelling method					
Utley and Shorrock [6]	1978	19,650 × 10^3^	Dwelling	13.6°C	
	1996	23,492 × 10^3^		16.1°C
	2006	25,285 × 10^3^		17.8°C

**Figure 1:**
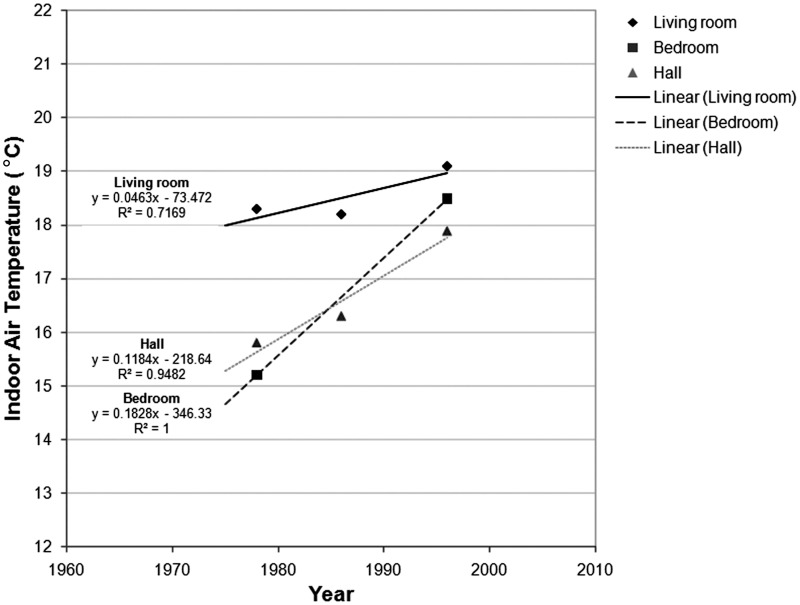
Mean winter indoor air temperatures trends based on national household surveys in the United Kingdom; data are obtained by daytime spot measurements (1978–1996) (Data sources: Hunt and Gidman [21]; DETR [22]).

Methodological and metereological differences across the various studies make it difficult to compare the data longitudinally. Nonetheless, if the comparison is limited to spot measurement monitoring studies, as illustrated in Figure 1, the evidence suggests that the average living room temperature has been increasing with a rate of 0.4°C per decade (from 18.3°C in 1978 to 19.1°C in 1996). A higher increasing rate is observed in bedroom temperatures (1.8°C per decade, from 15.2°C in 1978 to 18.5°C in 1996). Clearly, this indicates the impact of central heating penetration in the UK residential sector.

**Figure 2:**
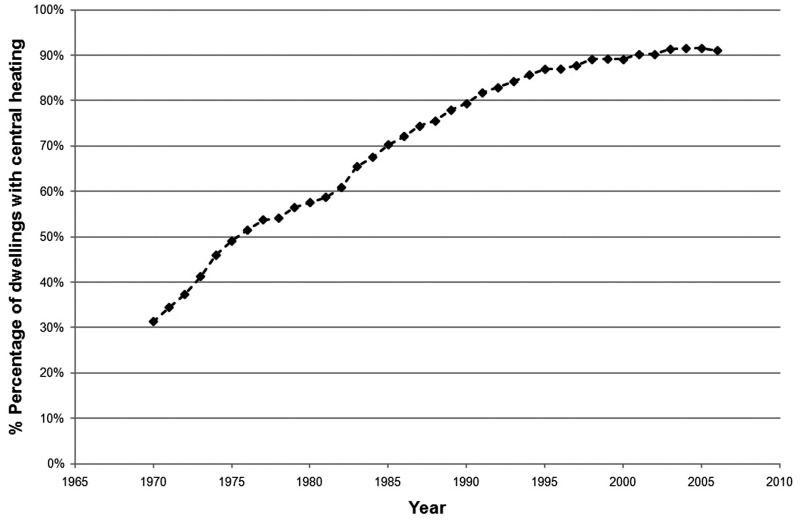
Central heating penetration in UK dwellings (1970--2006) (Data source: Utley and Shorrock [6]).

As shown in Figure 2, 91% of UK homes were served by central heating in 2006 compared to only 31% in 1970. It is important to note at this point that some of these measurements (half of them in the case of the Hunt and Gidman survey, [21]) may have been undertaken during the daytime when the sleeping spaces would have been commonly unheated and unoccupied. As a result, the bedroom temperature data should be treated with caution.

**Figure 3:**
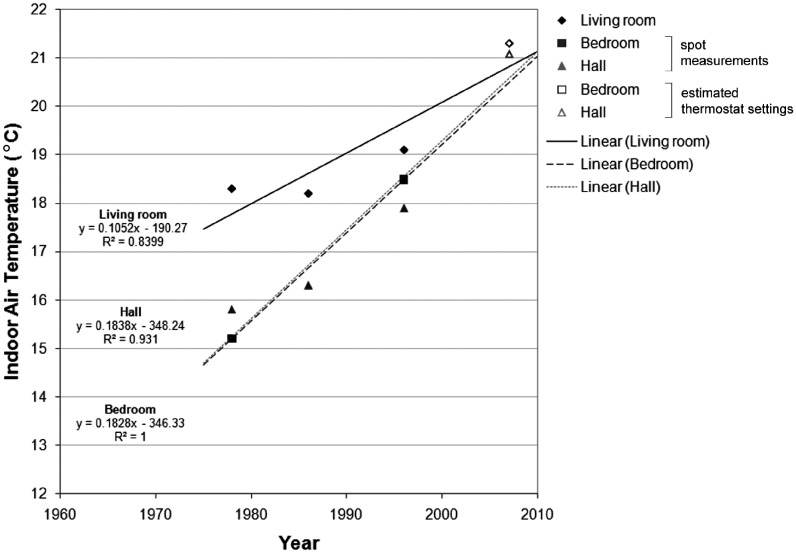
Mean winter indoor air temperatures trends based on national household surveys in the United Kingdom; data are obtained by daytime spot measurements (1978–1996) and estimated thermostat settings (2007) (Data sources: Hunt and Gidman [21], DETR [22], Shipworth et al. [16]).

If spot measurements are combined with more recent data based on estimated thermostat settings (Figure 3), the increasing temperature rate is sharper: 1.0°C per decade in living rooms (from 18.3°C in 1978 to 21.3°C in 2007). If we combine measurements in halls (a good proxy of mean dwelling temperature) with estimated mean dwelling thermostat settings, the calculated increase is 1.8°C per decade (from 15.8°C in 1978 to 21.1°C in 2007). Such a comparison, however, may lead to significant errors, taking into account that thermostat settings data are not directly comparable to spot measurement data.

**Figure 4:**
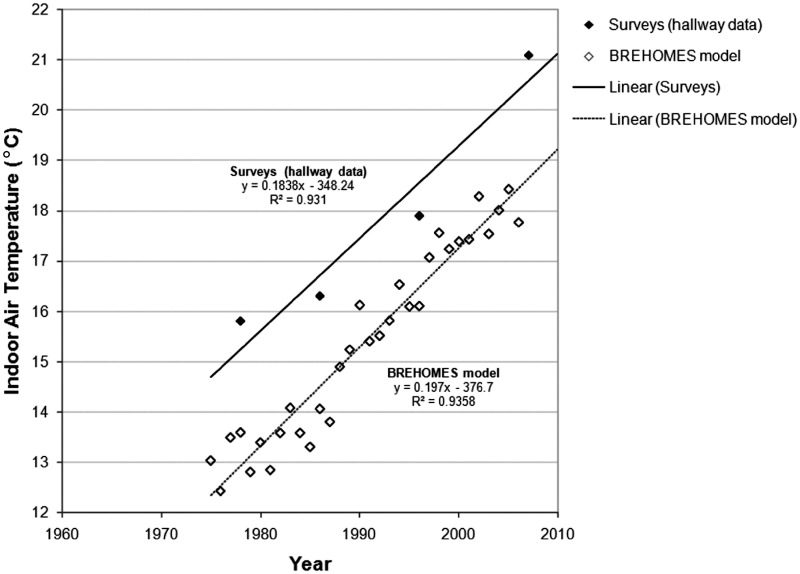
Comparison of mean dwelling winter indoor air temperature trends in the United Kingdom: national household survey data vs. modelled data; survey data are obtained by daytime spot measurements (1978–1996) and estimated thermostat settings (2007) (Data sources: Hunt and Gidman [21], DETR [22], Shipworth et al. [16]), modelled data are obtained by the BREHOMES model (Data source: Utley and Shorrock [6]).

Interestingly, the observed trend in survey data appears to match the trend emerging from the modelled data. As demonstrated in Figure 4, the increasing trend of 1.8°C per decade in surveyed halls compares well with the mean dwelling temperature increasing trend of 1.6°C per decade as calculated by the BREHOMES model. As suggested by this comparison, the size of relative changes can be estimated with more confidence than absolute figures.

### Other Industrialised Countries

Limited data from statistically representative samples of national domestic stocks exist pre-1970s. A series of “reported or measured” average indoor temperatures in nine countries of the industrialised world in the years following the energy crises of 1973–1974 and 1979 were presented in a study by Schipper et al. [24]. They reported an overall *decline* in indoor temperatures as a result of increased fuel prices. The differences are perceived to be the product of cultural differences as well as differences in prices and marginal utilization costs in the various countries (1979–1981). With the exception of Japanese households that maintained a mean dwelling temperature of 13–15°C in 1979, in the majority of the countries examined (Denmark, France, Germany and Italy) mean dwelling temperatures were within the range 17–20°C. The lowest range (16–18°C) was observed in Norway in 1981 and the highest average temperature (21°C) in Sweden in 1982. There is also indirect evidence of a similar behavioural change that took place in the United States in the 1970s chiefly fuelled by the energy crises [13,24]. The evidence consists of two household surveys: (a) a 1984 study of 1,700 houses and (b) the United States Residential Energy Consumption Survey (RECS), a national area-probability sample survey of 4,000 houses in 1981, as quoted in [13]. The existing data highlighted that the fuel price increases were reflected on indoor temperature decreases during winter and increases during summer as well as a rise in sales of automatic thermostats. Price elasticities of energy appear to have increased in magnitude in the early 1980s compared to the 1970s, although they seem to have decreased again after the mid 1980s. The short run price elasticity of energy was estimated to range between 0.00 to −0.16 in the late 1970s, which indicated that demand was relatively inelastic. According to aggregate dynamic model estimates, this value decreased to between −0.15 and −0.50 in the early 1980s but increased to between −0.03 and −0.35 in the mid-1990s [25]. It needs to be borne in mind, however, that, in conjuction with energy price increases, the reported space heating demand reduction was partly driven by the geographical shift of the U.S. metropolitan population towards the warmest South and West states that has been occurring since the 1960s [26,27].

Nonetheless, indoor temperatures appear to have increased postcrises. According to a comprehensive study of domestic indoor temperatures in 144 houses carried out during the winter and early spring of 1982 in Sweden by the Swedish Institute for Building Research [28,29], average temperatures as high as 21.8°C were recorded in multifamily and 20.4°C in single-family dwellings. A trend of increasing indoor temperatures and number of regularly heated rooms that took place in Norway throughout the 1970s and early 1980s was also reported [30].

**Figure 5:**
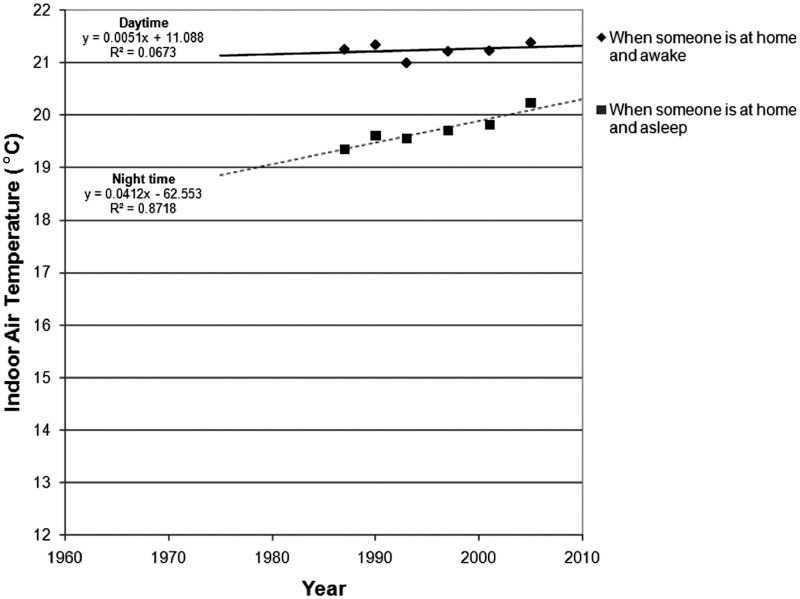
Mean winter indoor air temperature trends based on national household surveys in the United States; data based on self-reported thermostat settings (1987–2005) (Data source: EIA [32]).

Similarly, the decreasing winter indoor temperature trend in the United States was reversed within only 3 years, from 1984 to 1987 [13]. RECS data signify trends of rising winter internal living room and bedroom temperatures from the 1980s onwards [31] despite an overall decline in energy consumption for space heating, which is attributed to the increased efficiency of the building fabric and heating systems. Data on winter indoor temperatures were provided indirectly by means of self-reported thermostat settings (Figure 5). A general rising trend was recorded despite a slight decrease observed in 1996. Daytime dwelling temperatures “when someone was at home” remained fairly constant across the years (rising slightly from 21.2°C in 1987 to 21.4°C in 2005). A significant increase of approximately 0.5°C, on the other hand, was observed in temperatures “when someone was at home and asleep” (from 19.3°C in 1987 to 20.2°C in 2005). The data shown in the graph, however, exclude self-reported values of thermostats being off or missing data. It is, thus, likely to overestimate actual desired comfort levels.

Despite not being nationally representative, a series of studies conducted in Asian countries illustrate trends of increasing energy consumption and achieved winter and summer thermal comfort levels. A study in Seoul, Korea monitored indoor temperatures and occupant control behaviour of cooling and heating systems and subsequently compared the study output with the results of earlier studies carried out 25 years ago [32]. The study included 24 houses in summer, 6 houses in autumn and 36 houses in winter. It was demonstrated that the comfort temperature has increased in the heating period and decreased in the cooling period during the last 25 years. The mean indoor temperature was 27.5°C in summer, 23.7°C in autumn and 23.0°C in winter. Another survey of 240 Chinese houses located in three large cities (Beijing, Shanghai and Harbin) during the winter from 1998 to 2000 [33] found large temperature deviations between cities, which are mainly attributed to different heating systems and occupant choices. The mean indoor temperature is around 15°C in Shanghai where air conditioning is used for space heating and occupants tend to wear heavy clothing, compared to 20°C in Beijing, which is characterised by a high central heating penetration. A more recent survey of 76 houses in nine Chinese cities [34] demonstrated that the mean temperature of living room and bedroom remained stable between 18 and 20°C in Harbin, Urumqi, Beijing and Xi’an where houses were served by central heating systems. The inter-room temperature difference was also quite low. In contrast, much lower mean temperatures of living room and bedroom between 10 and 17°C were observed in cities where central heating is less common.

### Limitations

There are many limitations and sources of uncertainty associated with the evidence presented above:

*a. Spot measurements:* The uncertainty in the findings of Hunt and Gidman [21] and DETR [22], mainly arises from the fact that they adopted a spot measurement approach rather than temperature logging at a high temporal resolution (e.g. hourly) during consecutive days. Given that the spot measurements were predominantly carried out during the daytime when the majority of bedrooms were unoccupied, it is expected that higher levels of uncertainty are assigned to the reported bedroom temperatures, which may be underestimated. In addition, the proportion of changes in indoor conditions, which might have been due to variations in outdoor conditions cannot be accurately estimated.

*b. Self-reported settings:* Occupant self-reported values, such as the ones extracted from the US RECS surveys, should be treated with caution. In general, the thermostat settings are not necessarily representative of the mean thermal conditions occurring in a dwelling. Moreover, according to a previous U.S. study [35] actual recorded temperatures might be up to 1.1°C warmer than reported thermostat settings. The UK study by Shipworth et al. [16] also demonstrated that the mean thermostat setting estimated from loggers was more than 2°C higher than the corresponding mean respondent reported value.

*c. Model estimates:* Although model-generated estimates of absolute values, such as BREHOMES [6], are not as reliable as field evidence due to the inherent uncertainties of assumptions involved, they are significant in that they may highlight underlying trends. As was made clear by the authors, the absolute year-to-year values of these temperatures cannot be quoted with as much confidence as estimates of the extent of the rise. It has been suggested that the “reconciliation process” performed within the model to infer demand temperatures based on top-down energy statistics is a major source of uncertainty [15].

Whilst the limitations discussed above make it difficult to compare data and accurately estimate the size of historic changes in indoor domestic temperatures, data analysis does suggest an upward trend.

## Climate Change and Future Projections

### Climate Change

Due to climate change, it is likely that outdoor ambient temperatures will increase, thus reducing heating demand in the winter and increasing cooling needs in the summer. The way a building in a given region responds to cold and heat stress is influenced by a wide range of mostly socioeconomic region-specific structural indicators [36]. As the responses to cold and heat are different even for the same region, it is possible that increases in cooling demand will not always be offset by reductions in heating needs. The majority of studies examining the impact of climate change on the indoor thermal performance of buildings during the heating season have used the degree day approach: it is assumed that occupants will try to achieve the same indoor temperature levels (usually specified as a base temperature of 18°C) irrespective of outdoor conditions [37–43]. It is likely, however, that the population of previously heating dominated countries in the Northern hemisphere will shift their winter and summer thermal preferences towards the upper end of the comfort range, that is, similar to the temperatures in which Mediterannean populations feel comfortable, to reflect increasing external ambient temperatures. As a result, preferred indoor temperatures might be even higher in the future. Of the studies reviewed, only one study [36] addressed this issue by introducing a moving threshold of base temperature but their analysis remains at a theoretical level and was not applied for a specific region. No quantitative estimates of the increases in winter indoor temperatures due to climate change can therefore be provided at this stage.

### Energy Efficiency Refurbishments and the “Take Back” Factor

**Figure 6:**
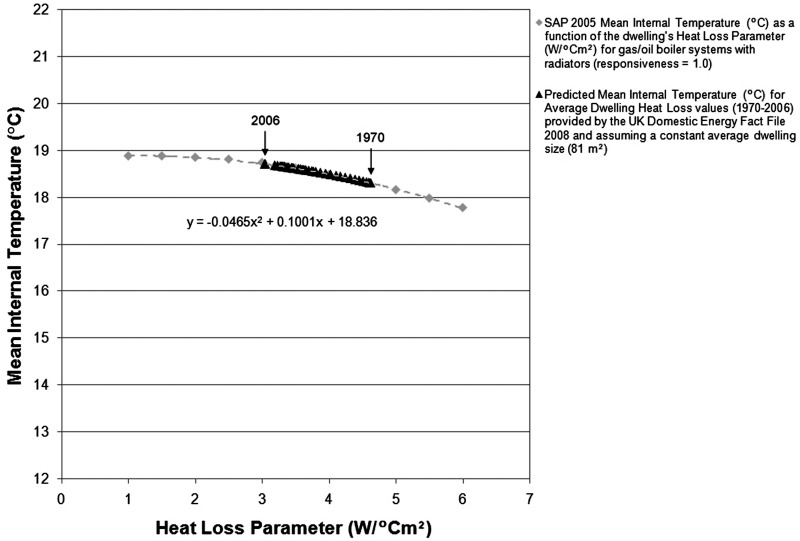
Mean internal temperature in a typical UK dwelling as a function of Heat Loss Parameter (Data sources: Utley and Shorrock [6]).

**Figure 7:**
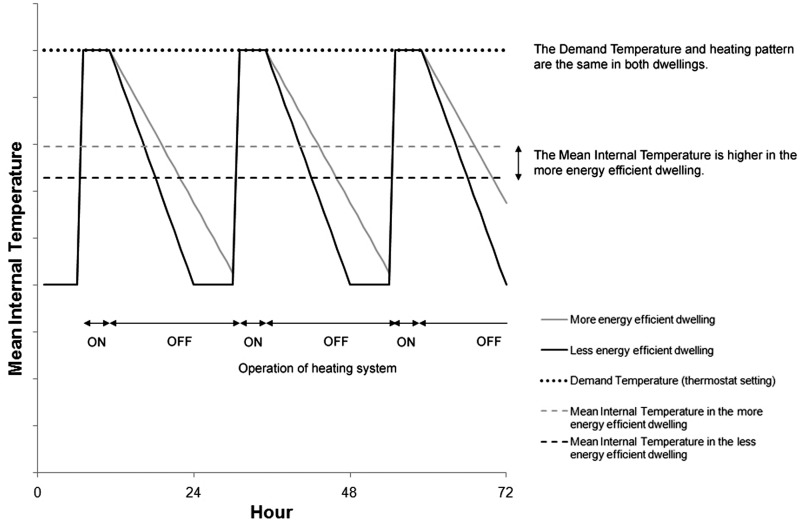
A schematic diagram of daily fluctuations in demand temperatures (thermostat set points) and mean internal temperatures as a function of the energy efficiency of the building envelope and heating systems.

To combat the dual threat of climate change and energy shortages, domestic building envelopes will become increasingly energy efficient in the future. As demonstrated in Figure 6, all other factors being equal, changes in the heat loss characteristics of the building envelope alone may be responsible for a significant increase in mean internal temperatures. This is futher exemplified in the schematic illustration of Figure 7; even if *demand temperatures* and *heating patterns* remain constant in the future, the more energy efficient dwellings will tend to cool down at slower rates than the less efficient structures. As a result, the *mean internal temperatures* in the former are likely to be higher compared to the latter. This suggests that even if people do not demand higher thermostat set points in the future, they may be subjected to higher internal temperatures partly due to living in more airtight environments or using more efficient heating systems.

Furthermore, demand temperatures are also likely to increase. Several studies have revealed that energy efficient retrofits, especially in fuel-poor households, are often used to improve indoor comfort conditions rather than reduce space heating fuel consumption (the “take back” or “comfort factor” [44]). It has been estimated that if energy retrofit works are carried out in an average income UK household with a mean internal temperature of 16.5°C, only 70% of the energy efficiency benefit will result in reduced fuel demand and 30% will be used to increase indoor temperatures [45]. This figure increases to 50% for a low-income household with mean dwelling temperatures of 14°C. The authors of this study suggested that the benefit of energy efficient improvements are likely to translate to energy savings in dwellings with whole house temperature above 20°C.

The Warm Front longitudinal study [46,47] was carried out during two consecutive heating seasons (2001–2002 and 2002–2003) in 1,372 mostly low-income (and therefore not nationally representative) households of mainly young families or elderly people in five cities in England pre- and post-energy efficient interventions. Living room and bedroom temperatures were monitored at half hourly intervals. The authors demonstrated that fuel-poor households that received both heating and insulation measures maintained the daytime temperatures 1.6°C higher in the living room and night time temperatures 2.8°C higher in the bedroom dwellings compared to pre-intervention conditions.

Another study [48] reported winter thermal comfort levels achieved pre- and post-thermal efficiency interventions in 100 UK households, which were “broadly representative of the national distributions” of buiding type and socioeconomic status. The sample of the households participating in the study were split into “priority” (mostly low-income and/or fuel-poor households recruited via the Warm Front scheme) and “non-priority” groups. Temperatures were measured at half-hourly intervals in living rooms, kitchens and main bedrooms. A mean dwelling temperature increase of approximately 0.6°C (from 19.2°C to 19.8°C) as a result of insulation upgrades was reported. It was demonstrated that only 60% of the calculated reductions in energy use of 629 kWh/day were actually obtained; the remaining saving costs were “taken back” as an increase in indoor thermal comfort.

Monitored temperature and energy consumption in 15 energy efficient dwellings in Milton Keynes were obtained in 1989–1991 and 2005–2006, as part of the Carbon Reduction in Buildings (CaRB) research project [49]. Mean temperature increased from 19.9°C to 20.1°C in living rooms but decreased from 19.7°C to 19.3°C in main bedrooms. Although the living room temperatures in middle- and high-income band households had not changed significantly, low-income households had increased their living room temperatures by approximately 1°C.

A nationally representative study of indoor temperatures and energy consumption was carried out in 400 homes in New Zealand between 1999 and 2005 (the Household Energy End-use Project, HEEP, [50]). The results in these newly built houses demonstrated a trend towards greater warmth in summer and winter. By comparing the internal summer temperatures in houses of different construction age bands, the authors estimated that the mean living room temperature is increasing by 0.25°C per decade of construction age. During summer, mean daytime living room summer temperature in post-1990 dwellings exceeded 20°C, with the average temperature equal to 23°C. During winter, living rooms in newer houses (built from 1978 onwards) were 1°C warmer on average and bedrooms were 1.3°C warmer.

### Fuel Prices and Thermal Comfort Adaptation

**Figure 8:**
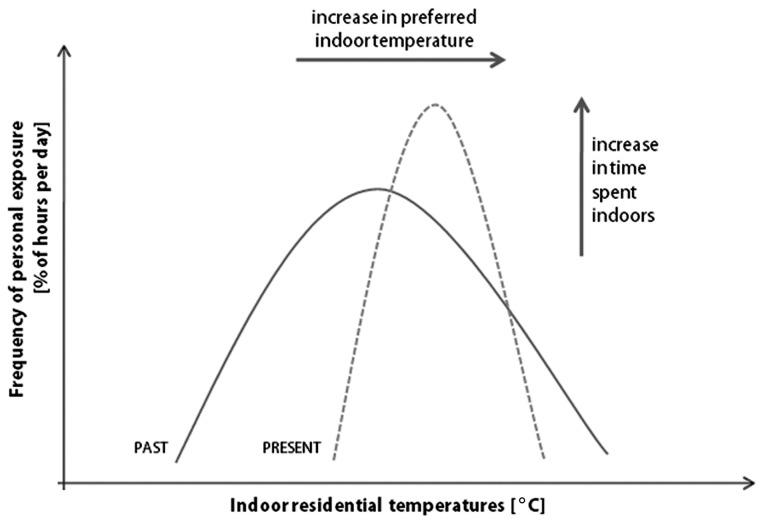
A schematic diagram of potential past and present distributions of personal exposure to indoor residential temperatures in heated-dominated countries with a westernised lifestyle.

This review has given an account of the overall increasing trends of indoor temperatures during the heating season worldwide and has investigated potential driving factors of this change. In terms of future comfort projections, two main scenarios are outlined based on the current literature, which mostly reflect the ongoing debate between the Fanger’s deterministic heat balance thermal comfort model [51] and Humphreys’ adaptive thermal comfort approach [52]:

*a. Continuing upward trends followed by stabilization at a high temperature due to saturation effects*: Meyer [53] argues that once people are accustomed to a high level of comfort, they are not willing to compromise. As a result, the human adaptability to thermal conditions is bound to become narrower in the future. The thermal comfort temperature is expected to lie within ranges specified by engineered thermal comfort chamber studies and perhaps this saturation limit will converge around the world towards “Western” standards [1,6]. Different authors have different views of the indoor temperature upper limit specification. In the worldwide context, this temperature is expected to be 21–22°C [6,54]. For the United Kingdom, this temperature is expected to be 19–20°C [6,55] under a business-as-usual scenario. In a recent publication by the Department of Energy and Climate Change (DECC), “2050 Pathways Analysis” [56], two out of four future scenarios of space heating demand in the United Kingdom included an increase in household demand temperatures within the range of +0.5°C and +1.5°C by the 2050s, compared to the rather low baseline modelled winter average of 17.5°C in 2007 provided by the Domestic Energy Fact File [6]. But there is also a significant trend towards heating the whole house due to the penetration of central heating and current projections estimate that by 2050, air-conditioning will be installed in half of all homes in England and Wales [6]. The schematic diagram in Figure 8 illustrates the possible change in the future winter comfort distributions: Not only *absolute desired winter indoor temperatures* may *increase* but also the *temperature ranges* in which individuals worldwide feel comfortable may become *narrower*.

*b. Downward trends linked to increased thermal comfort adaptability as a result of environmental awareness and higher energy prices:* Many authors [1,55] claim that there is still a significant potential for behavioural change. In their extensive review of comfort theories and future trajectories, Chappels and Shove [1] maintain that once we accept that thermal comfort is a sociocultural construct, we should be able to reconfigure social norms towards more sustainable practices. For instance, there is evidence that occupants tend to be more tolerant with low energy/passive heating, cooling and ventilative systems [57,58], the so-called forgiveness factor [59]. In the past, there have been examples of such behavioural shifts, such as the consumer adjustment and rise in automatic thermostat sales that took place in the United States during the 1970s–1980s when energy prices increased [13]. DECC [56] has examined two scenarios of reduced household demand temperatures within the range of −0.5°C and −1.5°C by the 2050s, compared to the baseline modelled winter average of 17.5°C in 2007 [6].

## Impact of Changes in Indoor Residential Temperatures on Weight Gain

It has been argued that increased exposure to thermoneutral conditions and the associated decreased exposure to mild seasonal cold as part of a Western lifestyle might be a contributing factor to weight gain [17,18,20]. Several experimental studies in controlled environments [60–66] have demonstrated that human energy expenditure increases in response to mild cold exposure and there appears to be a graded association between energy expenditure and ambient temperatures. This observation is of particular interest given that the temperature range examined in these studies (15–28°C) is similar to the range of temperatures experienced by occupants in domestic environments. A question that has not been addressed in these experimental studies, however, is how far the variation in energy expenditure at different temperatures might be reduced by behavioural factors in a more naturalistic setting, since the majority of the studies reviewed standardised participants’ food intake, clothing and activity levels. Whilst food intake reduces at higher temperatures, there is also evidence from animal studies that the availability of highly palatable and energy dense food may override the usual temperature-related compensatory adjustments in consumption [67]. This is particularly relevant in the context of industrialised countries where food is not only easily available but also energy dense.

In recent years, developments in the understanding of mechanisms of human thermogenesis and the role of Brown Adipose Tissue (BAT) have led to a renewed interest in the energy expenditure side of the energy balance equation [68]. BAT is a tissue which is, uniquely, able to expend energy in response to homeostatic requirements of the body, producing heat through cellular combustion. Present in large quantities in small mammals and human newborns, it was thought to be metabolically insignificant in adult humans, although recent studies have led to a reassessment of its importance, identifying active BAT in large proportions of adults [69–71]. BAT development and retention is induced by chronic cold exposure [72,73] and acutely activated in response to cold ambient temperatures [74–76]. It has also been shown to be subject to seasonal fluctuation [77,78].

Reduced exposure to cold may, therefore, have a dual effect on energy expenditure. First, since thermogenic capacity (and notably the development and retention of BAT) is stimulated by cold, an increase in time spent in conditions of thermal comfort may lead to loss of BAT and reduced thermogenic capacity. Second, more time spent in a thermal “comfort zone” reduces the frequency and/or duration of occasions on which cold-induced energy expenditure is initiated.

Based on published sources, it can be concluded that a causal link between reduced cold exposure and positive energy balance leading to adiposity is plausible [17,18,20]. To assess the magnitude of this effect, however, evidence of decreased energy expenditure needs to be examined in conjunction with estimates of the long-term changes in indoor ambient temperatures. For instance, Dauncey [62] estimated the potential impact of exposure to mild cold on weight loss by considering energy expenditures (EE) of 7716 and 8258 kJ/day measured in a chamber study at respectively 28°C and then 22°C. The conclusion was that “assuming other factors such as energy intake and external insulation to be equal, and that adipose tissue with an energy density of 25 MJ/kg is the major body component to be affected, then in 10 years, if these subjects had experienced mild cold for only 10% of each year they would have had, on average, an 8 kg loss in body-weight”.

A similar calculation can be applied by considering the estimated change in historic UK residential temperatures, and applying the relevant energy expenditure extrapolated from chamber studies. Warwick and Busby [63] estimated energy expenditures at 20°C and at 28°C as, respectively, 9.2 and 8.8 MJ/day. From the review of literature, this study has the smallest rate of change in EE following changes in temperatures, since it allowed participants a choice of clothing but prescribed a standardised activity and diet. Assuming no threshold effects, and applying the rate of change in EE from Warwick and Busby to the temperature changes likely to have occurred in the UK housing stock, it is possible to estimate the likely weight loss, which would occur if energy intake and activity levels were equal. The table below illustrates the potential weight gain associated with temperature changes as indicated from spot measurements in 1978 [21] and in 1996 [22], which were selected as being the most comparable and comprehensive. The calculations are partly dependent on the length of exposure to indoor residential conditions. Hence the table shows the predictions for exposures of 10, 8 or 6 h daily (over 18 years).

Data from Table 2 could be compared with the average weight gain of the UK population in the relevant timeframe. Unfortunately, currently available data on the average body weight and prevalence of obesity in the United Kingdom are available only from 1993 onwards. During that period, according to the Health Survey for England [79], the average weight of an adult person has been increasing at a rate of 0.3 kg per year, from 72.4 kg (SE = 0.12) in 1993 to 76.9 kg (SE = 0.17 in 2008). Whilst these data may not be immediately comparable with the estimates provided in Table 2 (e.g. different time scales, individual versus population level), the comparison suggests that the figures in Table 2 may be an overestimation – confirming that at present there is insufficient information to address the many sources of uncertainty and inaccuracies in the data used for Table 2 calculations. First, in a real-life context, activity levels and food intake would not be controlled as they were in the Warwick and Busby study [63]. Second, although spot measurement data shows the biggest historic thermal change in bedrooms, predictions associated with bedrooms are likely to be overestimated since temperature measurements were taken during the day whilst some bedrooms might have been heated at night. Furthermore, EE during sleep might be different from EE measured whilst at rest. It is also difficult to establish effects for different lengths of exposure to different residential environments (i.e. living rooms, bedroom). While temperatures in hallways are often considered to be representative of average values in dwellings, it is difficult to assess whether an average value could be meaningfully used in this context. Also, the rate of change in EE with changes in temperature differs across individuals: since chamber studies examine a small sample of male healthy individuals, wider population studies are needed. Finally, the calculations in the table above assume no significant temperature threshold effects in the rate of EE change. Although the experimental chamber studies suggest a graded association over thermal ranges which are relevant to UK residential environments, this has not been demonstrated outside controlled environments.

**Table 2:** Estimates of potential weight gain for an individual exposed to indoor temperature changes comparable with historic changes in UK domestic indoor temperatures

Location in dwelling	Average spot temperature measurements^a^ (°C)	Potential weight gain rate over the 18 year-period from 1978 to 1996 (kg/year)
			1978	1996	Exposure to residential environment: 10 h daily	Exposure to residential environment: 8 h daily	Exposure to residential environment: 6 h daily
Bedroom	15.2	18.5	1.0	0.8	0.6
Halls	15.6	17.9	0.7	0.6	0.4
Living Room	18.3	19.1	0.2	0.2	0.1

a Temperature data: 1978 data from the Hunt and Gidman study [21]; 1996 from the DETR EHCS [22].

## Discussion and Conclusions

The present review set out to summarize the literature on indoor temperature changes that have been observed in recent decades in industrialised countries. Potential implications of such changes in indoor climatic conditions were considered, such as the potential influence of decreased exposure of humans to seasonal cold on body weight gain.

Whilst methodological differences across studies make it difficult to compare data and accurately estimate the size of historic changes in indoor domestic temperatures, data analysis does suggest an upward trend, particularly in bedrooms. In the United Kingdom, for example, an increase of up to 1.3°C per decade in mean dwelling indoor temperatures in winter may have occurred from 1978 to 1996. However, the magnitude of these changes depends to a large extent on the thermal properties of the various national building stocks, as well as the fuel price regime of each country and outdoor temperature variations over the years. Also, the historic variations in indoor winter residential temperatures might have been further exacerbated in some countries by a temporary drop in indoor temperatures due to the 1970s energy crisis, as well as by more recent changes in the building stock (e.g. take back factor associated with energy efficiency refurbishment).

Changes towards a more sedentary indoor lifestyle, increased thermal comfort expectations, more efficient building stocks and rises in external temperatures due to climate change are all likely to further sustain an upward trend in internal winter temperatures. This phenomenon may be followed by reduced human adaptability to thermal conditions and by stabilisation due to saturation effects. On the other hand, some authors outline a different scenario characterised by downward trends in indoor temperatures linked to increased thermal comfort adaptability as a result of environmental awareness and higher energy prices.

The correlational evidence that links a decrease in the amount of time humans are exposed to mild seasonal cold and decreases in energy expenditure and adaptive thermogenesis is presented in detail elsewhere [20]. A case study providing a quantitative estimate of the effects of the observed changes in internal temperatures in UK houses on weight gain demonstrates the high level of uncertainty associated with these estimates, stemming not only from methods of collecting indoor temperature data but also from the use of estimates of changes in energy expenditure from chamber studies, which fail to take account of the clothing, diet or activity level adjustments that may take place in response to temperature changes in everyday life.

This review sought to find evidence of changes in the indoor domestic temperatures to which *individuals are exposed,* and their potential link with body weight gain. The indoor dwelling temperature might function as a good proxy for indoor temperature comfort levels but it is not necessarily the best representation of exposure levels. There are many other confounding factors that need to be examined to build a coherent image of current trends. A number of potential future directions of research are outlined below:

*a. Measure of change and threshold effects:* So far, indoor temperature trends have been expressed as the absolute change in mean temperature values indoors. With regard to potential linkage to obesity trends and health impacts, temperature excursions or the length of exposure might be equally important. Relative change within a given period of time will need to be quoted in conjunction with absolute values. It has been argued, for instance, that people will tend to spend more time indoors owing to the increased use of Information and Communication Technologies (ICT). Although the amount of time spent on indoor versus outdoor activities varies a lot across industrialised countries, the amount of time spent on indoor leisure has been increasing steadily from the late 1990s onwards in both the United Kingdom and the United States [80]. Additionally, a sharp fall of time spent on outdoor activities was observed during the 1990s. Moreover, further research is needed on possible threshold effects (e.g. temperature and energy expenditure, temperature and behavioural adaptations etc.), particularly outside the context of controlled chamber studies.

*b. Nondomestic environments:* The present work was limited to the examination of residential spaces despite the fact that people spend a considerable amount of their time working or commuting.

A further study with additional focus on nondomestic environments is suggested.

*c. Personal exposure:* The study of past exposure is limited to data on *room* conditions. Future research should refocus from the average temperature conditions in buildings to measuring the overall personal exposure of an *individual* in both domestic and nondomestic environments*. Personal exposure profiles* for “average individuals” who are representative of given socioeconomic groups of the population could be built. Sensors fixed to the person rather than the building could report on the actual exposure levels of these individuals in terms of both frequency and duration. A key question is to what extent this exposure has changed across the years as people tend to spend an increasing proportion of their time in temperature-controlled environments (offices, transport etc.). The impact of changes in human demographics on demand temperature (especially with regard to an ageing population, health status and vulnerability) should also be considered.

*d. Population studies:* Future research should also include large population samples, where conditions such as food intake and clothing adjustment are not controlled for, potentially leading to a wider variation in temperature-driven energy expenditure changes.

If sufficient evidence is provided for a link between increases in ambient temperatures and health impacts such as increases in obesity at the population level, it would be a key finding for both public health and building energy professionals. Not only it could inform strategies aiming to fight the “obesity epidemic” but it could also be associated with significant energy co-benefits as a result of reduced space heating demand in line with the global warming mitigation imperative to reduce the building sector’s CO_2_ emissions.

In summary, we have found that, although some evidence for a trend of increasing demand winter temperatures can be observed in existing building stock survey data, the generalisation of this trend to the entire stock is associated with high levels of uncertainty due to the scarcity of data and methodological caveats associated with data collection. Potentially, however, the mean internal temperature in winter may increase in the future solely due to energy fabric improvements and a rise in external ambient temperatures. Importantly, further chamber and population studies are needed to assess the possible links between changes in indoor temperatures and obesity. In addition, these links need to be investigated in light of the current trends in air-conditioning uptake, which would impact upon summer indoor temperatures.
